# The heart of the matter: modeling HIV-associated cardiovascular comorbidities in nonhuman primate models

**DOI:** 10.3389/fcimb.2025.1556315

**Published:** 2025-04-22

**Authors:** Vansh Khurana, Rodica Radu, Matthew J. Feinstein, Cristian Apetrei, Ivona Pandrea

**Affiliations:** ^1^ Department of Pathology, School of Medicine, University of Pittsburgh, Pittsburgh, PA, United States; ^2^ Internal Medicine Department, “Grigore T. Popa” University of Medicine and Pharmacy, Iasi, Romania; ^3^ Cardiology Department, Cardiovascular Diseases Institute “Prof. Dr. George I. M. Georgescu”, Iasi, Romania; ^4^ Division of Cardiology, Department of Medicine, Northwestern University Feinberg School of Medicine, Chicago, IL, United States; ^5^ Division of Infectious Diseases, Department of Medicine, School of Medicine, University of Pittsburgh, Pittsburgh, PA, United States; ^6^ Department of Infectious Diseases and Microbiology, Graduate School of Public Health, University of Pittsburgh, Pittsburgh, PA, United States

**Keywords:** simian immunodeficiency virus (SIV), human immunodeficiency virus (HIV), HIV comorbidities, antiretroviral therapy (ART), cardiovascular disease, hypercoagulation

## Abstract

With the advent of antiretroviral therapy (ART) that effectively suppresses HIV replication, and reduced AIDS progression, the clinical spectrum of HIV infection has dramatically changed. Currently, the people living with HIV (PLWH) who receive ART have a nearly normal prognostic of survival, yet they still experience higher morbidity and mortality than age-matched uninfected subjects. The higher risk of death in PLWH is linked to persistence of residual systemic inflammation and T-cell activation. These factors contribute to accelerated aging and higher incidence of HIV-associated non-AIDS conditions, thereby presenting new diagnostic and therapeutic challenges. This new shifting paradigm of HIV infection associates a higher incidence of cardiovascular disease (CVD), such as stroke, acute myocardial infarction and sudden cardiac death, in stark contrast to the reduced incidence of opportunistic infections. The incidence of acute myocardial infarction and coronary disease is several folds higher in PLWH than in the general population. Study of United States (US) death certificates listing HIV infection shows that the deaths from CVD doubled between 1996 and 2006. CVD will become an even more prominent comorbidity considering that more than 50% of PLWH in the US are over 50 years old, an age that more frequently associates CVD, and cardiovascular complications are more frequent in urban African-Americans and Hispanics, which are disproportionately affected by HIV. Therefore, reducing the overall risk of these complications will become the primary challenge in the management of chronic HIV infection. Not surprisingly, the REPRIEVE trial showed a substantial benefit of statins to PLWH, and the current guidelines include statin administration to PLWH. Nonhuman primate (NHP) models for the cardiovascular comorbidities associated with HIV are currently available and their use for testing new therapeutic approaches aimed at countering the effects of hypercoagulability and CVD is discussed. Their use can be of tremendous help to understand the etiology, pathophysiology, and the determinants of CVD in PLWH, which are currently poorly understood. Use of the NHP models could help in dissecting the relative contribution of the virus, behavioral factors, and ART to cardiovascular risk, having the potential to help us establish new strategic approaches aimed at controlling HIV-related CVD.

## 1 Introduction

Human immunodeficiency virus (HIV) remains a major public health problem worldwide, continuing to affect millions, particularly in developing countries, where treatment is less readily available and the clinical management of persons living with HIV (PLWH) is difficult. Antiretroviral therapy (ART) has emerged as the primary and standard treatment for HIV, significantly improving lifespan and general quality of life for PLWH (Deeks et al., 2015). However, the burden of the HIV epidemic remains high, and virus complete suppression is only achieved in a fraction of subjects even in the ART era, so that for every new person who starts ART, two new people become infected (Antiretroviral Therapy Cohort Collaboration, 2008). Furthermore, even when successful suppression of viral replication is achieved, ART does not completely restore immune integrity, nor does it fully eradicate HIV-1 (Policicchio et al., 2016; Cai and Sereti, 2021). Persistent residual systemic inflammation and T-cell immune activation can be observed in PLWH on ART, and life expectancy is not fully restored in ART-treated PLWH (Gabuzda et al., 2020; Cai and Sereti, 2021). Despite viral suppression, HIV infection is associated with accelerated aging, and continues to be linked to a wide range of comorbidities (Pandrea et al., 2015; He et al., 2017). These comorbidities, ranging from gut dysfunction to cardiovascular complications, are strongly associated with the residual inflammation and T-cell immune activation that persist under ART (Deeks et al., 2013; Cai and Sereti, 2021).

Cardiovascular complications, such as coagulopathy, atherosclerosis, arterial disease, myocarditis, myocardial infarction, and stroke, are among the most prevalent comorbidities associated with both ART-treated and untreated HIV/SIV infection (Pandrea et al., 2015; Gabuzda et al., 2020; de Camargo Vicioli and de Souza, 2024; Paternò Raddusa et al., 2024). The aging of the PLWH population further intensifies the risks for this group, as older individuals are generally more susceptible to cardiovascular events (He et al., 2017; de Camargo Vicioli and de Souza, 2024).

Chronic inflammation is pathognomonic for progressive HIV/SIV infections (Pandrea et al., 2008b), it is absent in nonpathogenic SIV infections in natural hosts (Pandrea et al., 2006; VandeWoude and Apetrei, 2006; Pandrea et al., 2008b; Pandrea et al., 2008a), and is triggered by multiple determinants: (i) persistently increased interferon responses (Harris et al., 2010; Sandler et al., 2014); (ii) preferential depletion of the Th17 cells, which preserve mucosal integrity (Brenchley et al., 2008; Favre et al., 2009); (iii) damage to the mucosal barrier, followed by microbial translocation (Brenchley et al., 2006b; Estes et al., 2010; Brenchley and Douek, 2012; Kristoff et al., 2014; Raehtz et al., 2020); (iv) coinfections with other pathogens (i.e., cytomegalovirus) (Freeman et al., 2015); and (v) ART toxicity and other drug-related risk factors (Deeks et al., 2013). Of these, damage to the gastrointestinal (GI) tract barrier and microbial translocation are among the most important pathogenic determinants of HIV/SIV disease progression (Brenchley et al., 2006b; Brenchley et al., 2006a; Barrenas et al., 2019; Raehtz et al., 2020; Apetrei et al., 2023), and a key catalyst for the development of HIV-associated comorbidities.

Endothelial dysfunction, hypercoagulation, and anemia are particularly critical factors in the pathogenesis of cardiovascular disease for PLWH, contributing to the increased morbidity and mortality observed in this population (Pandrea et al., 2022; Reno et al., 2022). However, the precise mechanisms through which HIV infection triggers these adverse cardiovascular outcomes are not yet completely understood, despite cardiovascular disease being recognized as a major cause of death in PLWH and SIV-infected macaques (Kuller et al., 2008; Pandrea et al., 2012).

As a complement to conventional clinical research, several animal models have been utilized for studying HIV pathogenesis. Nonhuman primates (NHPs), particularly SIV-infected rhesus (RMs) (Policicchio et al., 2016) and pigtail macaques (PTMs) (Pandrea et al., 2015), share a comparable, albeit not identical, genetic, and physiological profile to humans, allowing for the modeling of HIV infection through the use of an SIV infection. The effectiveness of ART has been tested and confirmed in these NHPs, facilitating studies that investigate the dynamics of SIV reservoirs necessary for a cure (Policicchio et al., 2016). NHPs were also extensively used for the study of prevention strategies, such as vaccine and microbicide testing and, more recently, pre-exposure (PEP) and postexposure prophylaxis (PrEP) strategies (Van Rompay, 2012; McNicholl, 2016; Martins and Watkins, 2018; García-Lerma et al., 2022; Counts and Saunders, 2023; Mayer and Allan-Blitz, 2023; Symmonds et al., 2024).

The use of NHPs for studying cardiovascular disease predates their application to the cardiovascular comorbidities associated with HIV/SIV infection. Thus, NHPs have been instrumental in demonstrating the role of diet in atherosclerosis and other cardiovascular disorders (Sasahara et al., 1994; Colman et al., 2009; Simmons, 2016). High-fat and high-cholesterol diets have been used to induce key indicators of human cardiovascular pathology, including plaque formation, arterial stiffening, endothelial dysfunction (Sasahara et al., 1994; Mattison et al., 2014; Zeng et al., 2015), as well as metabolic dysregulation, a critical risk factor for CVD (Zhang et al., 2011; Harwood et al., 2012). These translational studies demonstrated that NHPs can manifest pathologies and clinical signs of CVD, while sharing key risk factors with humans, thus providing valuable histological and pathological groundwork for investigating how HIV/SIV infection contributes to these conditions.

Meanwhile, extensive research in NHP models has characterized the innate immune system, including monocytes/macrophages and natural killer cells, independently of SIV infection (Wu et al., 2014; Ortiz et al., 2015; Wu et al., 2018; Rahmberg et al., 2024). These studies provided a robust foundation for the study of inflammatory mechanisms involved in the pathogenesis of cardiovascular comorbidities (Johnson et al., 2021). Although the use of NHPs for modeling HIV/SIV related cardiovascular comorbidities is relatively recent, these models can be instrumental in assessing the discrete mechanisms of these complications, their intricate relationships with chronic inflammation and immune dysregulation and their contribution to other HIV/SIV-associated comorbidities (Pandrea et al., 2015).

The scope of this review is to explore the current position of NHPs, particularly RMs and PTMs, as models for the study of cardiovascular comorbidities associated with HIV/SIV infection. We will discuss current treatments and potential therapeutic strategies, as well as potential research directions aimed at mitigating cardiovascular risk in PLWH. We will also address limitations and challenges associated with NHP use for studying HIV-related comorbidities, providing a holistic review of their current utility as a model for cardiovascular disease in PLWH.

## 2 The central role of chronic systemic inflammation and T-cell immune activation to HIV/SIV infection

Chronic inflammation and immune activation, both critical factors contributing to cardiovascular disease in HIV and SIV infections (Shen and Frenkel, 2004; Reno et al., 2022; Trøseid et al., 2024), are central to HIV/SIV pathogenesis. Persistent immune activation is triggered by viral persistence within the host (either as integrated proviral DNA or as antigens) (Dirajlal-Fargo and Funderburg, 2022; Lichterfeld et al., 2022). Multiple studies have shown that ART alone cannot eradicate HIV from an infected individual (Lau et al., 2021; Lichterfeld et al., 2022). Virus persistence and its homeostatic proliferation induce a state of chronic inflammation, even in PLWH that have been suppressed for decades (Jacobs et al., 2019; Deeks et al., 2021; Lau et al., 2021), leading to a variety of pathological changes that increase cardiovascular risk (Pandrea et al., 2012; Paiardini and Müller-Trutwin, 2013; Schechter et al., 2017).

Left untreated, HIV and SIV infections elicit a drastic and chronic inflammatory response, primarily caused by continuous viral replication. This replication causes persistent epithelial dysfunction, directly damaging the gut integrity, leading to increased levels of translocation of microbes and microbial products from the intestinal lumen to the general circulation (Hao et al., 2015), resulting in hyperactivation of various immune cell populations (Brenchley et al., 2006a; Brenchley and Douek, 2012; Sandler and Douek, 2012; Le Hingrat et al., 2021). The impact of damage to gut epithelial integrity in driving microbial translocation was confirmed by showing microbial leakages in the vicinity of breaks within the epithelial lining (Estes et al., 2010). Microbial translocation was documented in mucosal tissues (lamina propria, gut-associated lymphoid tissue, mesenteric lymph nodes), as well as in distant lymph nodes and the circulation (Brenchley et al., 2006b; Estes et al., 2010; Hao et al., 2015). These microbial products eventually reach the circulation and fuel local and systemic inflammation, along with macrophage activation (Brenchley et al., 2006b; Pandrea et al., 2008a; Estes et al., 2010). CD4^+^ T cell depletion and hyperactivation cause excessive release of inflammatory cytokines associated with active infection, activating several downstream cascades that may potentially trigger cardiovascular comorbidities, including cyclical release of inflammatory markers and immune cells (Le Hingrat et al., 2021). HIV-associated inflammation disrupts endothelial function, reducing nitric oxide production and leading to impaired vasodilation (Marincowitz et al., 2019; Mokoena et al., 2024). Inflammatory cytokines further hyperactivate endothelial tissue, upregulating adhesion molecules and subsequently promote leukocyte migration adhesion, leading to further vascular infiltration and inflammation. These factors lead to a damaged vasculature, inducing endothelial dysfunction and accelerating the pathogenesis of atherosclerosis (Higashi et al., 2009). In conjunction with arterial disease, myocardial dysfunction and other severe complications may occur in PLWH left untreated for sustained periods of time (Ntusi, 2017). Interestingly, recent studies have shown that subclinical coronary artery plaque can be associated with levels of biomarkers of both systemic (e.g., IL-6) and atherosclerotic plaque (e.g., lipoprotein-associated phospholipase A2 inflammation). These associations occur in the absence of other traditional risk factors (Ntsekhe and Baker, 2023). Therefore, in such instances, traditional cardiovascular risk indexes might be normal.

Another prominent mechanism causing a chronic inflammatory state in PLWH is the depletion of CD4^+^ T cells, particularly within the gut-associated lymphoid tissue (GALT) (Lau et al., 2021; Le Hingrat et al., 2023). The current paradigm of HIV/SIV infection pathogenesis is that the depletion of CD4^+^ T cell subsets involved in gut homeostasis (i.e., Th-17 CD4^+^ T cells), combined with gastrointestinal tract inflammation and T-cell activation, drives the deleterious consequences of HIV infection. CD4^+^ T cells, together with myeloid cells, are killed and release inflammatory cytokines (Hasegawa et al., 2009; Laforge et al., 2011), such as IL-1β and IL-6, thus creating an inflammatory environment (Doitsh et al., 2010; Doitsh et al., 2014). Meanwhile, the loss of cells that are involved in maintaining epithelial integrity, homeostasis, and antimicrobial defense, i.e. IL-17 and IL-22-producing cells (Raffatellu et al., 2008; Klatt et al., 2012b; Xu et al., 2012), complement the virus’ action in damaging the gut and increasing the loss of epithelial integrity, enteropathy and microbial translocation (Heise et al., 1994; Kewenig et al., 1999; Estes et al., 2010). Systemic inflammation and immune activation trigger a vicious cycle in which new CD4^+^ T cells migrate to the gut, increasing the number of susceptible cells and reactivating proviruses in latently-infected cells (Doitsh et al., 2014). Newly produced viral proteins and viruses can further boost inflammation, tissue damage, and microbial translocation.

Translocated microbial products also trigger an inflammatory cascade *via* recognition of toll-like receptors on immune cells (Brenchley and Douek, 2012), initiating signaling events that activate transcription factors regulating various inflammatory genes. Factors such as NF-kB are activated and subsequently promote the production and release of more inflammatory mediators, including chemokines, cytokines, and adhesion molecules (Liu et al., 2017). These molecules attract more immune cells to inflammatory sites, perpetuating the inflammatory response and causing progressive immune cell infiltration into tissue. Within the cardiovascular system, increased presence of immune cells can exacerbate vascular inflammation, leading to potential development of atherosclerotic plaques over time (Marincowitz et al., 2019). Myocardial infiltration with immune cells results in myocyte death and myocardial fibrosis (Ntsekhe and Baker, 2023).

These inflammatory pathways promote the development of a hypercoagulable state, a well-established risk factor for thromboembolic events such as myocardial infarction and ischemic stroke (Obare et al., 2024). Elevated levels of coagulation factors have been observed in both PLWH/SIV-infected macaques and are closely linked to chronic inflammation and endothelial dysfunction. Procoagulant factor fibrinogen increases in response to inflammatory cytokines and promotes both platelet aggregation and thrombi formation (Graham et al., 2013). D-dimer, a marker of fibrin degradation, is positively associated with clotting activity and correlates with markers of endothelial dysfunction as well (Graham et al., 2013). These incidences suggest a relationship between inflammation, NET-osis, endothelial damage, and coagulopathy in PLWH, a relationship which has been demonstrated in SIV-infected NHP models as well (Pandrea et al., 2012; Schechter et al., 2017).

### 2.1 HIV-associated cardiovascular comorbidities

HIV/SIV infections are associated with a myriad of comorbidities spanning multiple physiological domains, and stemming from persistent inflammation and immune dysregulation. These comorbidities continue to affect PLWH even under ART, and include cardiovascular disease, liver complications, metabolic disorders, lung and kidney diseases, hypercoagulation, and neuroinflammation (Gabuzda et al., 2020; de Camargo Vicioli and de Souza, 2024; Majonga et al., 2024; McCutcheon et al., 2024; Paternò Raddusa et al., 2024; Pericàs et al., 2024; Sarkar and Brown, 2024). While complications associated with HIV/SIV infection are numerous, this paper specifically examines current research and associated challenges within the cardiovascular system.

Cardiovascular disease is one of the most prevalent comorbidities in PLWH (McCutcheon et al., 2024). Even on ART, PLWH are at a higher risk of these comorbidities, which include peripheral/coronary artery disease, pulmonary arterial hypertension, and hypercoagulation (McCutcheon et al., 2024). These conditions are precursors and significant risk factors for severe acute cardiovascular events, including myocardial infarction and ischemia, which are potentially fatal and more prevalent in PLWH than in the general population (Nazari and Feinstein, 2024). In PLWH, the main factor behind cardiovascular disease is represented by the heightened levels of chronic inflammation. Induced proinflammatory cytokines activate an inflammation-dependent pathway in endothelial cells lining vessels, leading to adhesion of monocytes to the endothelial lining and subsequent vascular inflammation (Marincowitz et al., 2019). Increased vascular permeability has also been observed as a consequence of this pathway, although the exact mechanism is not fully understood (Marincowitz et al., 2019). Endothelial dysfunction is a well-characterized precursor to atherosclerosis, as well as associated cardiovascular events, including subsequent PAD/CAD and possible myocardial infarction, ischemic stroke etc (Rajendran et al., 2013). While specific viral proteins are associated with endothelial dysfunction and can be reduced through ART, TNF-α and IL-6 have been reported as sources of dysfunction as well (Ridker, 2016). These markers continue to be elevated in PLWH on ART (Zhou et al., 2007) and are associated with microbial translocation from the gut to the bloodstream, highlighting a link between gut dysfunction and cardiovascular risk.

Metabolic disorders, including insulin resistance and diabetes, also have a higher incidence in PLWH on ART compared to HIV-uninfected individuals (Bourgi et al., 2018). Metabolic alterations are an additional catalyst for cardiovascular complications (Grundy et al., 2019). Inflammatory cytokines, which are upregulated in PLWH regardless of ART status, have been shown to induce insulin resistance through binding to elements within skeletal muscle, adipose tissue, cardiac muscle and subsequently interfering with insulin signaling mechanisms (Akash et al., 2018). TNF-α and IL-6 are two of several cytokines implicated in the development of insulin resistance, a precursor to type 2 diabetes and significant risk factor for cardiovascular disease. Both TNF-α and IL-6 are increased during chronic HIV/SIV infections (Aukrust et al., 1999; Borges Á et al., 2015).

Gut dysbiosis, which is frequent in HIV/SIV infections and involved in microbial translocation (Klase et al., 2015; Zevin et al., 2016), results in imbalances of beneficial bacteria, particularly those that produce short-chain fatty acids (SCFA). SCFAs have been found to have a role in many metabolic pathways; SCFAs stimulate glucose absorption, improve lipid metabolism and reduce fat synthesis/storage, and are involved in maintaining the insulin/glucagon balance for overall energy metabolism (He et al., 2020). Therefore, a decrease in SCFA-producing bacteria due to dysbiosis can catalyze metabolic dysregulation by both altering overall metabolism and promoting inflammation due to absence of anti-inflammatory effects. Metabolic disorders may be further exacerbated by adipose tissue dysregulation, which persists and may even be enhanced in PLWH on ART. In particular, adiponectin levels were lower in PLWH receiving ART (Kinlaw and Marsh, 2004), while leptin levels were found to be higher (Hikasa et al., 2023). Adiponectin and leptin are major regulators of metabolism as well as anti- and proinflammatory cytokines, respectively, indicating that metabolic complications in conjunction with elevated inflammatory markers are still an issue even in the presence of ART.

Meanwhile, coagulopathy is a well-defined risk factor for major cardiovascular events, including ischemic stroke, peripheral and coronary artery diseases, and myocardial infarction (Kuller et al., 2008; Pandrea et al., 2012; Reno et al., 2022; Hikasa et al., 2023). PLWH on ART not only display elevated markers of general inflammation, but also distinct markers of hypercoagulability, notably D-dimer (Perkins et al., 2023). Even if viral loads are suppressed with ART, persistent hypercoagulation (Dirajlal-Fargo and Funderburg, 2022) places PLWH at a higher risk for adverse cardiovascular conditions. Cardiovascular health may be further affected by other issues like endothelial damage to blood vessels, causing further vascular complications and coagulopathy (Perkins et al., 2023).

## 3 Current therapeutic approaches targeting the cardiovascular comorbidities associated with HIV infection

Numerous strategies are available for the treatment of cardiovascular disease in the general population. Historically, management of cardiovascular disease in PLWH typically consists of ART in conjunction with cardiovascular treatments aimed at ameliorating symptoms (e.g. ART + warfarin for lowering risk of thromboembolic events) (George et al., 2020; Sang et al., 2021; Seo et al., 2021). While ART is essential for controlling viral replication and restoring some immune function, it does not fully address cardiovascular risk linked to chronic inflammation and immune activation. Current ART regimens have been shown to induce inflammation, metabolic dysregulation, and adipose tissue remodeling in PLWH — all clear risk factors for cardiovascular health (Flint et al., 2009; Bailin et al., 2024). These findings are mirrored in NHP studies (Mausoleo et al., 2022; Webb et al., 2024). However, while the relations between ART use and immune/metabolic dysregulation are well established, their exact mechanisms are not fully understood. Consequently, standard care for PLWH often involves concomitant treatments directed at mitigating adverse effects and lowering cardiovascular risk. This section reviews the outcomes of various current treatments for managing cardiovascular comorbidities in PLWH. Additionally, we evaluate outcomes from SIV-infected NHP studies to identify gaps between translational and clinical research and highlight potential treatments requiring further investigation.

### 3.1 Statins

Statins are a complementary therapy of choice for PLWH, given the important immunometabolic alterations associated with HIV infection (Sáez-Cirión and Sereti, 2021), and a substantially increased risk of myocardial infarction in this population (Mocroft et al., 2010; Smith et al., 2010). In addition to significantly reducing blood cholesterol levels, statins have low-density lipoprotein cholesterol (LDL-C)-independent effects, including reductions in oxidative stress and anti-inflammatory properties; they reduce levels of C reactive protein, suppress synthesis of interleukins (IL-1β, IL-6, IL-8, IL-10), and inhibit T-cell activation (Choudhary et al., 2023). These pleiotropic actions can provide additional cardiovascular protection for PLWH (Kuller et al., 2008; Shah et al., 2018).

In terms of using statins for the primary prevention of cardiovascular disease, we rely on traditional cardiovascular risk calculators for general population (i.e., atherosclerotic cardiovascular disease, ASCVD risk calculator). For example, the ASCVD risk calculator includes: (a) age, sex, and race; (b) blood pressure and use of medications to treat high blood pressure; (c) cholesterol levels and use of statins to treat high cholesterol; (d) diabetes status; (e) family history of heart attacks or heart disease, especially before age 60; (f) history of aspirin therapy to lower the risk of heart problems; and (g) smoking history. However, the ASCVD risk calculator does not include inflammation as a risk factor, thus not reflecting the actual increased risk in PLWH, in whom persistent inflammation drives a higher likelihood of developing cardiovascular diseases compared to people without HIV (Shah et al., 2018). As such, the field could consider the experience with PLWH and include chronic inflammation as a major risk factor for cardiovascular disease.

The results of the Randomized Trial to Prevent Vascular Events in HIV (REPRIEVE) showed that pitavastatin calcium 4 mg/day (vs placebo) is an effective measure to prevent major cardiovascular events (MACE) in low-to-moderate CV risk (according to ASCVD) PLWH under stable ART (Grinspoon et al., 2023). The reduction in the incidence of MACE by 36% and of MACE or death by 23% over a median of 5.1 years was striking enough to prematurely terminate the REPRIEVE trial. Consequently, statin administration was incorporated into the latest Clinical Guidelines for the primary prevention of atherosclerotic CVDs in PLWH, aged 40–75 years (https://clinicalinfo.hiv.gov/en/guidelines/hiv-clinical-guidelines-adult-and-adolescent-arv/statin-therapy-people-hiv).

However, it remains unclear whether the protective effects of pitavastatin in PLWH can be generalized to other statins. Atorvastatin was also shown to reduce levels of activated T-lymphocytes in untreated PLWH, without any effect on HIV-1 RNA levels (Ganesan et al., 2011), suggesting that statin-induced metabolic remodeling may help control inflammation in PLWH. In PLWH on ART, a more realistic model for developed countries, rosuvastatin was shown to enhance CD8^+^ T-lymphocyte functionality and survival. The group for this study did not have any clinical indications for statin use either (Perdomo-Celis et al., 2024), suggesting some primary degree of prevention from rosuvastatin. These findings indicate that atorvastatin and rosuvastatin, in conjunction with ART, may help control inflammation, slow the progression of subclinical atherosclerosis, and prevent cardiovascular comorbidity, regardless of initial risk factors. In two clinical trials, treatment with a moderate-intensity statin (atorvastatin 20 mg/day) reduced the volume and vulnerability features of coronary artery plaques in ART-naïve PLWH (Lo et al., 2015). Additionally, rosuvastatin (10 mg/day) was associated with reduced progression of carotid artery intima-media thickness in ART-treated PLWH (Longenecker et al., 2016). Altogether, these results indicate that all statins have some beneficial effect in preventing cardiovascular disease in PLWH.

In NHPs infected with SIV, limited data exists for the broad range of statins that may be used, as preclinical testing was completed in other animal models. However, one study administered atorvastatin in a SIV-infected, untreated RMs to assess the effect of statin administration on pulmonary arterial hypertension (PAH) incidence. Results indicated that incidence of PAH was lower in statin-treated RMs; inflammatory mediators, including TNF-α, TGF-β, and MIP-1α, along with cell subtypes associated with PAH, were all decreased (Rabacal et al., 2019), supporting the role of statins in controlling inflammation.

While findings from these trials are promising, further studies are needed to determine the anti-inflammatory and cell dynamic effects of the wide range of available statins. Statins largely utilize the same mechanism, yet vary in potency and side effects; it is therefore necessary to investigate whether (a) particular statins have a greater anti-inflammatory effect in NHPs, humans, or both, and (b) particular statins have different (i.e., exacerbated or attenuated) effects when administered with or without ART. Furthermore, ART and statin coadministration has potential to induce novel adverse effects, including muscle symptoms and new-onset diabetes (https://clinicalinfo.hiv.gov/en/guidelines/hiv-clinical-guidelines-adult-and-adolescent-arv/statin-therapy-people-hiv), which must be investigated and monitored. Based on clinical trials evaluating statins in HIV patients receiving ART and their drug–drug interaction potentials, pitavastatin is considered safe, whereas atorvastatin and rosuvastatin may require dosage adjustments (https://clinicalinfo.hiv.gov/en/guidelines/hiv-clinical-guidelines-adult-and-adolescent-arv/statin-therapy-people-hiv). Lovastatin, fluvastatin or simvastatin should not be used concomitantly with ART because of interactions with HIV protease inhibitors and/or lack of sufficient clinical data (https://clinicalinfo.hiv.gov/en/guidelines/hiv-clinical-guidelines-adult-and-adolescent-arv/statin-therapy-people-hiv; Chastain et al., 2017). Despite limited data available for all statins for PLWH, human and NHP data converge on supporting the paradigm that statins, as a class of medication, reduce systemic inflammation and the consequent incidence of cardiovascular disease. Current guidelines recommend the integration of statins as an adjuvant therapy for PLWH, offering a degree of both primary and secondary prevention for a population with elevated cardiovascular risk. Specifically, (a) high-intensity statin therapy is recommended for individuals age 40–75 years with a high (≥20%) 10-year atherosclerotic cardiovascular disease (ASCVD) risk; (b) high-intensity statin therapy at the maximum tolerated dose is recommended for individuals aged 20–75 years who have LDL-C levels ≥190 mg/dL; and (c) at least moderate-intensity statin therapy is recommended for individuals aged 40–75 years with diabetes mellitus. In these patients, further risk assessment will be performed to evaluate the necessity for high-intensity statin therapy (https://clinicalinfo.hiv.gov/en/guidelines/hiv-clinical-guidelines-adult-and-adolescent-arv/statin-therapy-people-hiv).

### 3.2 Anticoagulants

**Figure 1:**
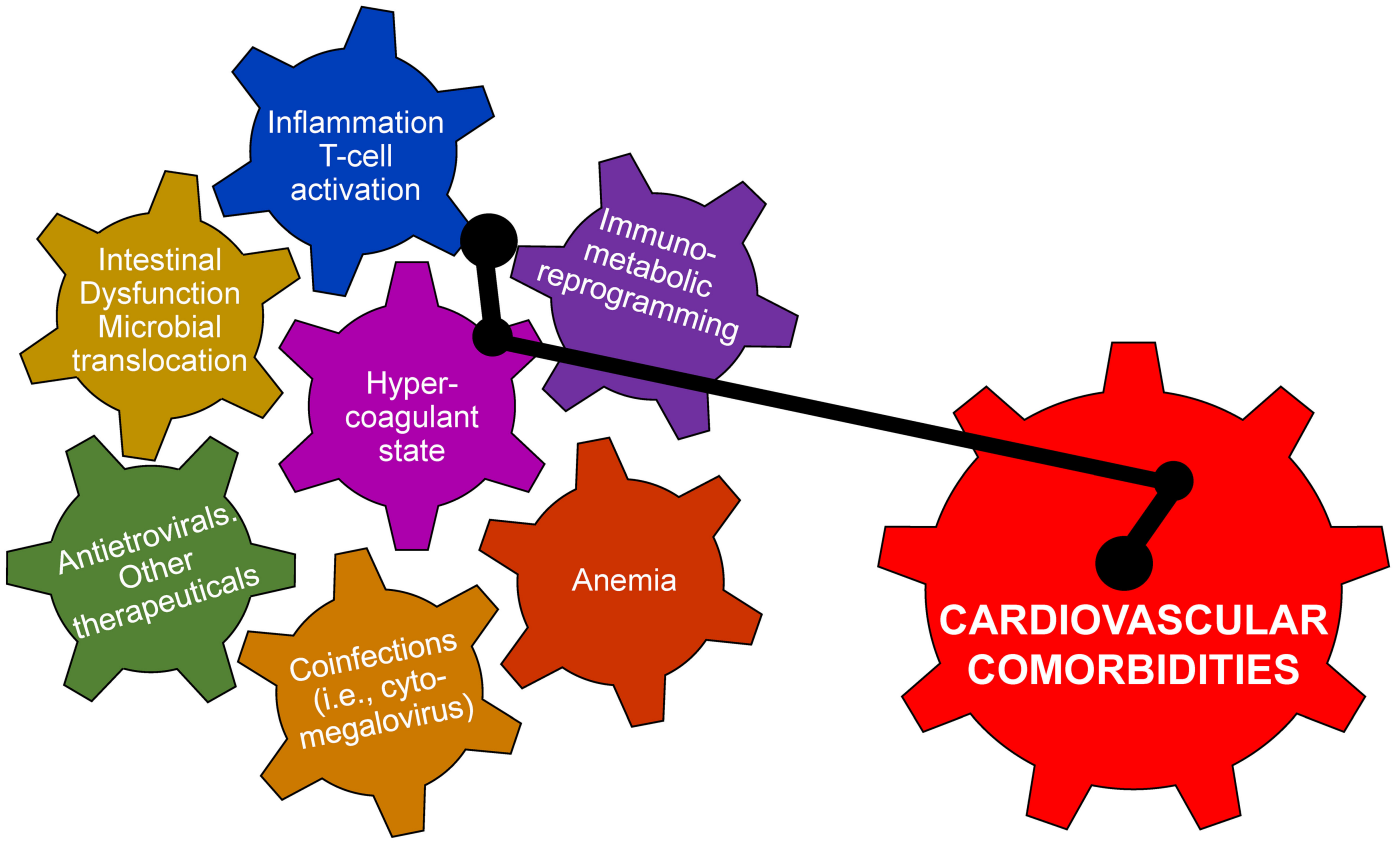
There are multiple pathogenic determinants of cardiovascular disease in persons living with HIV and in SIV-infected macaques. They are driven by viral infection and are interrelated and mutually potentiating. Altogether, their interactions can trigger the development cardiovascular disease.

Thrombotic events are also a cause for concern for PLWH, with venous thromboembolisms (VTE) being the third most common cardiovascular condition for PLWH (Zhang et al., 2022) and arterial thrombosis as another common condition (Perkins et al., 2023). PLWH display elevated procoagulation markers, including D-dimer, fibrinogen, and soluble CD163 (Kuller et al., 2008; Boulware et al., 2011; Burdo et al., 2013; Okello et al., 2016; Bryant et al., 2017). ART generally lowers plasma levels of coagulation and inflammation markers (Sereti et al., 2017; Cai and Sereti, 2021) compared to untreated infection, but above the preinfection levels. Meanwhile, PLWH on ART are still at a higher risk for hypercoagulation due to residual inflammation (Kuller et al., 2008; Cai and Sereti, 2021). The relationship between coagulation and inflammation is now understood as bidirectional, with inflammation markers activating coagulation cascades and coagulation markers inducing inflammatory pathways and gut dysfunction (Schechter et al., 2017; Cai and Sereti, 2021; Reno et al., 2022) (**Figure 1**). Nevertheless, the exact mechanisms linking inflammation, coagulation, and risk of thrombotic events largely remains unknown. Anticoagulant medications therefore continue to be investigated as treatments with a potential “bidirectional” effect, with the inhibition of procoagulation factors potentially attenuating inflammation and mitigating cardiovascular risk.

The potential use of anticoagulants and antiaggregants in PLWH warrants a higher level of consideration. Drug interactions between antiretrovirals and anticoagulant medications are both predicted through pharmacokinetics (Egan et al., 2014) and have been clinically reported (Liedtke and Rathbun, 2009; Welzen et al., 2011; Corallo et al., 2015), due to inhibition of CYP complexes for drug metabolism (Liedtke and Rathbun, 2009; Egan et al., 2014). In addition, anticoagulants pose an obvious bleeding risk. Clinical trials involving anticoagulants in conjunction with ART have been conducted, but exclusively for patients with existing indications for anticoagulant therapy to prevent more adverse cardiovascular events (ADVICE study group, 2018; Baker et al., 2020; Brazeale et al., 2023). Due to several contraindications and potential adverse reactions that are predicted and not confirmed (and vice versa), anticoagulants cannot be safely tested as a preventative or experimental therapeutic agent in clinical settings (Lomakina et al., 2023). As a result, the ability to study the specific relationship between hypercoagulation and inflammation is somewhat limited.

Very few studies have been published involving anticoagulants in NHPs, in general as part of their preclinical evaluation (Utoh et al., 2015; Honickel et al., 2017; Kouta et al., 2020; Poitout-Belissent et al., 2020; Wong and Quan, 2021). Even fewer studies are available that test the impact of anticoagulant administration in SIV-infected NHPs (Schechter et al., 2017; Carson et al., 2025). In the first study, an experimental tissue factor inhibitor (Ixolaris) was administered to SIV-infected PTMs at the time of SIV infection. PTMs treated with Ixolaris showed significantly reduced levels of inflammation and monocyte and CD4^+^ T-cell activation (Schechter et al., 2017). Tissue factor expression on circulating CD14^+^ monocytes decreased in Ixolaris-treated PTMs compared to untreated controls, with Ixolaris-treated PTMs also exhibiting reduced monocyte activation. Together, these results suggest that Ixolaris treatment had a beneficial effect in reducing immune activation and inflammation associated with SIV infection. Remarkably, Ixolaris administration significantly reduced plasma D-dimer levels and viral loads during acute and early chronic SIV infection, while also delaying disease progression (Schechter et al., 2017), indicating a clear effect of Ixolaris in improving the coagulation status and modulating SIV pathogenesis. These results may be of significance as they suggest that tissue factor inhibition may abrogate rapid progression in treated animals, likely due to the combined effect of reduced immune activation and inflammation, reduced hypercoagulability and a small reduction in plasma viremia (Schechter et al., 2017).

Since there is no FDA-approved tissue factor inhibitor, in a second study we evaluated the utility of apixaban (Eliquis) as an adjuvant to ART in reducing inflammation and hypercoagulation in SIV-infected RMs (Carson et al., 2025). Apixaban is a factor Xa inhibitor used to treat and reduce the risk of stroke, deep vein thrombosis and pulmonary embolism, and was selected for its ability to inhibitor Factor Xa, an early and therefore critical step within the coagulation and thrombin generation cascade. This study did not find any significant differences between apixaban-treated and control groups for CD4^+^ T-cell recovery in blood and tissues. Furthermore, apixaban did not significantly impact immune activation, inflammation or coagulation status in the SIV-infected ART-treated RMs. Meanwhile, apixaban-treated RMs experienced hemorrhaging issues throughout the follow-up (Carson et al., 2025). Given apixaban’s lack of effect on immune activation, CD4^+^ T-cell restoration, inflammation and coagulation — along with an increased risk of hemorrhaging — factor Xa inhibition does not appear a realistic option to reduce the incidence of cardiovascular events in aging PLWH (Carson et al., 2025).

In spite of these dichotomic effects of anticoagulants in improving the prognosis of SIV infection in NHPs, these studies hold real potential in addressing key questions regarding the utility of anticoagulant treatments in PLWH. The NHP model allows for a detailed characterization and more invasive studies to establish the impact of these treatments on coagulation status and cardiovascular risk. In humans, such studies could pose ethical challenges, as the risk of bleeding or potential drug interactions can frequently outweigh benefits for patients without a clear clinical indication for anticoagulant therapy. In contrast, NHPs potentially provide a more suitable platform for a more speculative or trial-and-error approach to investigation, without the potential ethical risk posed by human trials.

Nonetheless, based on results from our group and others, we anticipate that anticoagulant therapy is unlikely to achieve the same traction as statins for the primary prevention of cardiovascular comorbidities in PLWH. Several arguments contribute to this perspective: (i) the potential complications of anticoagulant use (e.g., bleeding) are more frequent and more severe than those typically seen in cholesterol-lowering drugs; (ii) anticoagulants have only shown a modest efficacy in normalizing coagulation parameters in PLWH and SIV-infected NHPs, suggesting that either standard dosing regiments may be insufficient for PLWH/SIV-infected NHPs, or that other mechanisms drive hypercoagulability in these individuals; and (iii) in contrast to statins, current anticoagulants have not demonstrated any notable impact in reducing inflammation in PLWH and SIV-infected NHPs, implying that additional or alternative pathways must be targeted to mitigate cardiovascular risk within these individuals.

### 3.3 Non-steroidal anti-inflammatory drugs

NSAIDs have long been studied for their ability to inhibit inflammation, making them a potential option in reducing cardiovascular risk (Bogaty et al., 2004). These drugs traditionally inhibit the cyclooxygenase (COX) enzymes – COX-1, COX-2, or both – subsequently reducing production of pro-inflammatory prostaglandins (Simon, 1999; Morita, 2002). However, in previous studies, the use of combined COX-1/COX-2 inhibitors was reported to cause adverse gastrointestinal effects and increase the risk of bleeding, due to COX-1 inhibition (Simon, 1999). Therefore, more current approaches involve the use of selective COX-2 inhibitors as potentially “safer” alternatives.

One of the most common NSAIDs for PLWH is COX-1/COX-2 inhibitor acetylsalicylic acid (ASA/aspirin), which has a dual action: anti-inflammatory and anti-platelet aggregation (Ogawa et al., 2008; Antithrombotic Trialists et al., 2009). Aspirin is also a unique inhibitor of nuclear factor kappa B (NF-κB), a transcription factor involved in cell proliferation and a central mediator of inflammation (Zheng et al., 2017). This inhibition is distinct from the mechanisms of other NSAIDs, and therefore aspirin may also directly interfere with the life cycle of HIV-infected cells (Chen and Stark, 2017; Mussbacher et al., 2019). The “baby aspirin, once a day” recommendation was once widely promoted amongst the general population, particularly the elderly, to prevent blood clots and cardiovascular events (Darraj, 2024). However, recent clinical guidelines have shifted away from this practice (Force et al., 2022), as randomized controlled trials (RCTs) have found that the risk of GI bleeding and hemorrhagic complications frequently outweigh the benefits of aspirin as a primary preventative agent (McNeil et al., 2018). Instead, lower-dose aspirin is now recommended as a secondary preventative agent for atherosclerotic disease once indications and risk factors are present (McNeil et al., 2018).

For studies involving PLWH on ART, the effects of aspirin as an anti-inflammatory and therefore preventative agent are inconsistent. One randomized controlled trial found that an 81 mg dose of aspirin reduced the levels of sCD14, a marker of immune/monocyte activation and inflammation (Mystakelis et al., 2023) that is also an independent predictor of disease progression and death in PLWH (Sandler et al., 2011). Similar results, in conjunction with decreases in CD38 and HLA-DR, were found in another study involving aspirin therapy in PLWH on ART for one week (O’Brien et al., 2013). However, another trial concluded that aspirin had no significant effect on any biomarkers of or T-cell immune activation, systemic inflammation, or coagulation — including sCD14 (O’Brien et al., 2017). Similarly, another study with a platelet-centered focus found that, while aspirin reduced platelet aggregation in PLWH on ART, it did not have a significant impact on lowering platelet activation markers such as P-selectin and PAC-1 (Marcantoni et al., 2022), both critical indicators of inflammatory pathways within the vasculature. Ambivalence in findings in PLWH reflects updated clinical guidelines for the general population, which state that “in primary prevention, aspirin use is more controversial,” while its role is “well established for secondary prevention of [atherosclerotic cardiovascular disease]” (Arnett et al., 2019; Force et al., 2022).

No literature is available for the use of aspirin in the NHP models, particularly to reduce hypercoagulability, yet this does not come as a surprise. Aspirin has been in clinical use for well over 100 years, predating HIV emergence and the animal modeling of AIDS. As a result, aspirin has shifted to clinical practice without preclinical testing. However, aspirin can still be tested in a controlled environment with an NHP model, potentially in conjunction with other experimental treatments.

A myriad of newer selective COX-2 inhibitors are available, developed with the goal of potentially lowering prostaglandin release while simultaneously avoiding inhibition of COX-1-mediated platelet aggregation (Capone et al., 2003). COX-2-activated monocytes, macrophages, and T-cell infiltrates have been observed in the hearts of some HIV-1-infected/AIDS patients, suggesting that COX-2 plays a role in inflammation and resulting cardiomyopathy (Prebensen et al., 2017). However, clinical studies examining COX-2 inhibitors as anti-inflammatory agents have produced mixed results. For example, etoricoxib, a highly selective COX-2 inhibitor, reduced the fraction of all T-cell subsets expressing CD38^+^ and decreased thrombin generation *in vitro* in ART-naïve PLWH (Prebensen et al., 2017). However, no significant impact on inflammation or immune response was found in the ART-treated PLWH arm, suggesting that efficacy of COX-2 inhibitors may be impacted by their association with ART (Prebensen et al., 2017). A less selective COX-2 inhibitor, celecoxib, was reported to also induce a reduction in the CD38^+^ expression by T cells in ART-naïve PLWH (Pettersen et al., 2011).

Selective COX-2 inhibitors were also minimally tested in NHP settings. Meloxicam is the only agent for which we have macaque data, but not in relation to SIV infection. In a brief 4-day study, meloxicam was compared to aspirin in regard to the balance between perioperative pain management and platelet aggregation as a risk factor for hemorrhage (Anderson et al., 2013). In contrast to aspirin, which significantly decreased platelet aggregation as expected, meloxicam did not significantly modify the levels of platelet aggregation compared to baseline values (Anderson et al., 2013). However, this study was limited in scope, as it only evaluated meloxicam’s tolerability in healthy RMs. The effects of meloxicam or other COX-2 inhibitors, in SIV-infected NHPs, on or off ART, have not been published; therefore, it remains unclear whether COX-2 inhibition, at varying levels of selectivity, could serve as a potential preventative anti-inflammatory agent for HIV/SIV-related cardiovascular complications.

### 3.4 Diet and nutrition

The impact of diet on HIV/SIV pathogenesis in both ART-treated and untreated individuals has not been investigated enough, compared to specific treatments or medications for comorbidities. In the general population, a high fat, “Western-style” diet (HFD) was reported to strongly associate gut dysbiosis and subsequent inflammation (Malesza et al., 2021), along with metabolic disturbances and subsequent dyslipidemia (Feingold, 2000). In PLWH, ART-treated individuals on an HFD exhibited significantly higher levels of proinflammatory and hypercoagulation biomarkers (Manzano et al., 2022) compared to a “Mediterranean” diet that limits cholesterols/lipids and emphasizes plants, grains, and protein-rich foods.

Results from SIV-infected NHPs paralleled clinical studies, with HFD-fed ART-naïve PTMs exhibiting significantly accelerated disease progression compared to a non-HFD group (He et al., 2019). SIV-infected PTMs within the study showed distinct signs of CVD, including myocardial cell infiltration, myocardial fatty infiltration, microthrombi, pericardial fibrosis, and early signs of atherosclerosis compared to the non-HFD group (He et al., 2019). African green monkeys (AGM), natural controllers of SIV that typically do not progress to AIDS, were also negatively impacted by the HFD, exhibiting increased activated CD4^+^ T cells, higher abundance of C-reactive protein (CRP), higher viral loads, and even progression to AIDS in one animal (He et al., 2019). These findings suggest a heightened state of systemic inflammation associated with SIV presence in conjunction with an HFD.

While the impact of an HFD in macaques on ART is still under investigation, early observations suggest that an HFD exacerbates disease pathogenesis even when viral loads are controlled (Brooks et al., 2023). NHP models therefore allow for further study of nutritional impact on cardiovascular health for PLWH within a more controlled environment, without the plethora of confounding factors that impact the human studies, and with a strict control of diets and doses. Such findings from NHPs suggest the need for potential dietary modifications in some PLWH to mitigate cardiovascular risk.

## 4 Use of NHPs to model HIV-associated cardiovascular comorbidities

While NHPs share a high degree of physiological and structural similarity to humans, significant limitations still exist with the simian model. Several challenges of using the NHP model will be subsequently discussed in this section, with an emphasis on the advantages and limitations of the use of NHPs to study the impact of cardiovascular comorbidities in PLWH (**Table 1**).

**Table 1:** Rationales for the use of nonhuman primate modeling of cardiovascular complications of HIV infection and possible future directions of research.

Aspect	Pros	Cons	Future Perspective
**Physiology**	Closely resembles cardiovascular and immune systems	Species-specific differences in immune response/disease progression	Integrate data from different species to simulate human condition
**Pathophysiological Modeling**	Replicates key CVD features (e.g. inflammation, hypercoagulability)	Accelerated models may not capture gradual disease onset (e.g. atherosclerosis) and its determinants	Development of models with realistic and prolonged risk factors
**Experimental Control**	Allows for controlled infections and invasive sampling at well-defined critical time points of infection, detailed analysis	Short follow-ups due to ethical boundaries and premature euthanasia	Optimize study design to extend observation, while preserving NHP welfare
**Therapeutic Testing**	Permits preclinical testing of interventions in controlled settings without behavioral risk factors	Limited preclinical data and variability in treatment response (e.g. pitavastatin vs. atorva/rosuvastatin)	Conduct further studies to fine-tune therapeutic strategies

Shortly after the emergence of HIV infection worldwide, NHPs infected with SIV were discovered to be a highly applicable and translatable model for studying HIV pathogenesis (Desrosiers and Ringler, 1989; Kleinman et al., 2022). NHPs continue to be an invaluable asset in HIV research due their close genetic relation to humans, which results in near-identical physiological and structural characteristics compared to humans (Estes et al., 2018; Kleinman et al., 2022). Furthermore, NHPs are outbred, which is a great advantage for the study of comorbidities in comparison with mice, which are inbred (Vierboom et al., 2005). More recently, the macaque model of HIV infection has been shown to be a valuable tool for the study of HIV-associated comorbidities (Pandrea et al., 2012; Pandrea et al., 2015; Estes et al., 2018).

Among the progressive models for HIV pathogenesis, RMs and PTMs have emerged as two of the most used models due to their similarities in immune response and disease progression compared to humans (Estes et al., 2010; Pandrea et al., 2015; Kleinman et al., 2022). The macaque model of HIV infection is considered one of the best animal models for any given disease (Estes et al., 2018), in which we can properly model both the pathogenesis of HIV infection, immune responses conferring protection to vaccination, and efficacy of new strategies for virus reactivation from latent reservoirs (Burton et al., 2012; Policicchio et al., 2016; Kleinman et al., 2022). However, there are also inherent limitations of the model; most notably, the inability to infect monkeys with HIV directly, and SIV being more closely related to HIV-2 than to HIV-1 (Apetrei et al., 2005; Kleinman et al., 2022). There are also differences in the pathogenicity of SIV infection among RM models, with RMs of Indian origin more consistently in progressing to AIDS, and RMs of Chinese origin exhibiting a relatively high rate of spontaneous control in up to one third of cases (Ling et al., 2002a; Ling et al., 2002b). Infection also appears to be slightly more pathogenic in PTMs than in RMs (Mandell et al., 2014), likely due to the fact that PTMs have higher baseline levels of systemic inflammation compared to RMs. This heightened inflammation may be attributed to a higher frequency of interleukin-17 (IL-17) production from producing (Th17) cells as a consequence of preexisting gut dysfunction (Favre et al., 2009; Klatt et al., 2010; Pandrea et al., 2012; Canary et al., 2013). Both models can be employed for the study of HIV-associated comorbidities, particularly cardiovascular disease (Klatt et al., 2012a; Pandrea et al., 2012; Pandrea et al., 2015). PTMs have more consistent and prominent increases in levels of hypercoagulation markers, including D-dimer, making them useful for studying both the longitudinal onset and management of cardiovascular comorbidities, and also offering potential for studies focused on the effects of chronic inflammation on cardiovascular health over time (Pandrea et al., 2015).

PTMs and RMs both exhibit immune decline when left untreated, with many but not all animals exhibiting cardiovascular system failure over time (Klatt et al., 2010; Klatt et al., 2012a; Mandell et al., 2014). Primary prevention treatments can be tested in these animals for their efficacy in curbing inflammation and preventing comorbidity development during early stages. This potentially translates to slower comorbidity development over time, or even early mitigation of some inflammation-associated comorbidities. However, while a more rapid disease progression and higher inflammation in PTMs offers utility for acute and early stages, these same characteristics limit the applicability of the model for chronic conditions seen in humans under ART. This holds particularly true not only for cardiovascular comorbidities, but for many longer-term comorbidities that develop over extended periods of time, including hepatic and neuroinflammatory disorders.

RMs also exhibit a heightened but slower and more controlled progression following SIV infection. This yields value when studying more chronic facets of HIV infection, including the effects of longer-term ART and the longitudinal onset of cardiovascular comorbidities. A slower disease progression allows for the extended study of impacts on levels of immune reconstitution, inflammatory response, and comorbidity development over time. As a more longitudinal model compared to PTMs, RMs are therefore better at explaining how more chronic, low-level inflammation can contribute to more gradual-onset disorders in general, including cardiovascular/metabolic disorders.

In the current scope of research on simian models, the selection of a PTM or RM model is essential for addressing research questions related to long-term/chronic effects vs. short-term/acute infection. To effectively model HIV pathogenesis in the human host, impact of ART and development of comorbidities, data from both PTM and RM studies must be integrated. This allows for the representation of both short-term inflammatory responses and chronic effects of low-level inflammation and ART, creating a more holistic model for the development of ART-related comorbidities in PLWH. However, integrating data from two separate species to simulate human infection is less ideal than a single model that simulates human infection; while nonhuman primates share similarities in DNA to each other and to humans, there are marked differences between species in immune and regulatory pathway expression, compromising the direct translatability of models to clinical settings. Following integration of findings from simian models, translational research with simian models in conjunction with human models is a crucial step that must be investigated extensively prior to clinical applications.

Models of natural SIV infection, such as AGMs infected with SIVagm, can also be employed to compare and contrast pathways leading to comorbidities. Natural hosts of SIVs that do not progress to AIDS demonstrate an exquisite ability to keep chronic inflammation and T-cell activation at bay, while maintaining baseline levels of coagulation biomarkers (Pandrea et al., 2012).

To effectively study HIV-associated cardiovascular complications, comparison and understanding of the differences between humans and NHP models is essential for a myriad of reasons. While similarities (e.g., vascular and gastrointestinal anatomy, markers of inflammation) are apparent between human and NHPs, differences in immune response — notably disease progression — still exist. Furthermore, ethical constraints often prohibit the investigation of very advanced stages of comorbidities in animal models, as in most instances they occur at the Institutional Animal Care and Use Committee (IACUC) protocol endpoints.

### 4.1 Development of atherosclerosis as the major limitation of the NHP model

Atherosclerosis and atheromatous plaques have been demonstrated in the RM model. Plaques in uninfected RMs were detected via ultrasound and identified in conjunction with inner arterial thickening (IMT), following the administration of a HFD for 96 weeks (Zeng et al., 2015). Due to the extended time required for the development of full atherosclerosis, precursor lesions were investigated. Arterial fatty streaks, which are precursors to atherosclerosis, were identified in a SIV-infected PTM model (He et al., 2019). While these precursors were successfully induced in NHPs using a HFD (He et al., 2019), several factors limit the direct applicability of findings to atherosclerosis/atheromatous plaques in humans and PLWH. One factor is that the HFD administered to NHPs in both studies contained approximately 300% fat content compared to the control diet, a level likely significantly exceeding realistic “Western” diet content. Within the uninfected RM study, this fat content was utilized to accelerate plaque formation within a relatively short study period of 96 weeks. In contrast, humans typically develop atherosclerosis over the course of many years, and a diet with drastic fat content administered for a shorter duration is likely not completely representative of the plaque formation and subsequent progression to atherosclerosis observed in humans.

This “accelerated” model in NHPs also cannot capture the multitude of factors that may contribute to atherosclerosis. In humans, variables including genetics, preexisting conditions (diabetes, hypertension, hypercholesterolemia, etc.), smoking, and lifestyle can all collectively contribute to gradual formation of atherosclerotic plaques (Sattelmair et al., 2011; Wirtz et al., 2017; Lechner et al., 2020). For PLWH, interaction of these well-classified factors over an extended period (years) can be linked to chronic inflammation and the endothelial dysfunction associated with atherosclerosis. In contrast, NHP studies such as those described above rely on a single, more exaggerated risk factor — extremely high dietary fat — to induce plaque formation more rapidly. These NHP study designs, while providing the ability to isolate at least one factor contributing to arterial disease, conversely may fail to capture the cumulative effects of multiple risk factors in PLWH that interact over several years.

Meanwhile, it is common practice to euthanize NHPs within 1–2 years to preserve quality of life. However, this practice also creates limitations on studying a full progression to atherosclerotic disease. For humans, atherosclerosis and plaques can persist for years before causing a major cardiovascular event (Nambi et al., 2010; Owens et al., 2012; Xing et al., 2021). While plaques and precursors have been successfully induced in NHPs, the short durations of these studies may not allow for the observation of more advanced plaque development, major changes in artery composition, or major alterations in plaque, including instability and potential rupture, which may also cause major cardiovascular events (Camare et al., 2017).

Therefore, while NHPs can be potentially used to model the specific mechanisms behind risk factors associated with atherosclerosis, they may fail to capture the gradual progression of arterial disease that occurs within humans and therefore PLWH. Future studies may benefit from the development of a model that incorporates several realistic risk factors over longer periods of time. However, this also presents an ethical challenge, as inducing these risk factors may significantly reduce quality of life for many animals involved.

### 4.2 Histological features of cardiovascular comorbidities in NHP

NHP studies permit a more invasive and detailed approach for documenting the manifestation of many physiological comorbidities, including — but not limited to — cardiovascular ones. Serial sampling of blood and tissues can be performed and combined with histological examination following ethical sacrifice of NHPs; this dramatically extends our clinical approaches, or permits correlating biomarkers of cardiovascular disease with specific lesions. In studies involving cardiovascular comorbidities, NHPs can be ethically and strategically sacrificed at appropriate times. This allows for the examination of the heart, vasculature (i.e., carotid thickness), and other tissues that can be involved in cardiovascular and metabolic disorders, including the liver. From here, pathological structures (or lack thereof) can be identified to indicate a particular study outcome. Lesions, infiltration, necrosis, etc. can all be identified post-necropsy, which is not possible in clinical settings.

However, a limitation inherent to NHP studies is their vascular system. Atherosclerosis and atheromatous plaques are central to a discussion of cardiovascular health and are a key factor in the pathogenesis of severe cardiovascular events. However, atherosclerosis and plaques are not uniformly distributed, and are more concentrated at certain structures within the vasculature (e.g., furcations, high shear-stress points) (Wasilewski et al., 2023). While NHP models do allow for the direct and detailed examination of selected arterial sites (coronary, brachial, aorta etc.), histologically surveying all arteries is logistically unfeasible. Techniques such as MRI and CT angiography (premortem) can be used, but these methods either lack the ability to detect more subtle pathological changes or are frequently unfeasible due to accessibility to investigator. Realistically, direct observation of plaques and atherosclerosis must be performed in areas determined by the researcher, which may not consistently provide a holistic view of disease within the vasculature.

Despite this limitation, pathological examination of tissues collected from NHP necropsies revealed a broad spectrum of cardiovascular abnormalities (Chalifoux et al., 1992; Eitner et al., 1999; Shannon et al., 2000) that recapitulated those reported in HIV patients (Pandrea et al., 2012; Ntsekhe and Baker, 2023). The pathologic evidence of CVD consisted of numerous thrombi present in the glomerular capillary loops and small arteries in the kidneys, resembling the renal thrombotic microangiopathy (TMA) described in PLWH. Similar changes were also observed in the small blood vessels of the intestine, lung, and brain (Pandrea et al., 2012). Interestingly, these TMAs appear to be specifically associated with pathogenic SIV infection, as they were absent in uninfected macaques and SIV-infected AGMs, which do not progress to AIDS (Pandrea et al., 2012).

### 4.3 Ethical/welfare concerns regarding presentation of comorbidities in NHPs

When researching comorbidities related to SIV or ART, ethical challenges become a significant issue, particularly in instances where early euthanasia is necessary. When modeling comorbidities under ART, particularly for chronic and slower-developing complications (e.g., atherosclerotic plaques, coronary/peripheral artery disease (CAD/PAD), liver damage and potential failure), balancing necessary data while accounting for animal welfare is a challenge. While dynamics for markers associated with certain disorders can be observed and outcomes extrapolated, data collection may be prematurely terminated prior to actual presentation of symptoms associated with comorbidities. Chronic disease progression and frailty can lead to a significant decline in quality of life for NHPs in studies, potentially requiring premature euthanasia to prevent suffering. For example, an animal may present with elevated D-dimer levels earlier in a protocol but require euthanasia due to reduced quality of life before experiencing any major cardiovascular event. This complicates longitudinal studies aimed at understanding the chronic effects and complications of ART, particularly for comorbidities that develop more slowly. Diseases like metabolic disorders, liver fibrosis, and CAD take years to develop and are often deeply interconnected. Investigators researching the scope of long-term comorbidities must balance the need for effective and conclusive data with the responsibility of maintaining animal welfare. Thus, premature euthanasia will continue to be an extremely sensitive consideration when studying long-term comorbidities; finding this balance is an ongoing ethical challenge in basic and translational science research.

## 5 Conclusion and future directions

NHP modeling of cardiovascular disease associated with HIV infection has real potential to improve our understanding of the pathogenesis of cardiovascular diseases and therapeutic approaches to tackle it — not only in PLWH, but also in the general population. Association between experimental therapeutics and the more invasive sampling possible in NHPs allows us to better understand the association between risk factors and cardiovascular lesions, thereby improving diagnostics, treatment and the quality of life of PLWH. Use of these large animal models, which are genetically close to humans and outbred, can enable us to perform complex studies aimed at exploring various pathogenic axes, such as the gut-heart-brain axis, and assess how changes at one of these sites can trigger pathologic events at different levels of these axes. Moreover, the use of the animal model can enable us to perform invasive studies aimed at assessing the impact of lesions occurring at various levels of these axes at different levels. For instance, we can experimentally damage the gut and explore the impact on the cardiovascular or central nervous system. Similarly, we can experimentally induce myocardial infarctions and assess their effects on the systemic levels of inflammation — or, conversely, assess how preexisting levels of inflammation influence the severity of cardiovascular diseases.

Despite the fact that, due to ethical limitations, rapid disease progression and shorter follow-up periods, advanced cardiovascular disease cannot be always achieved in NHPs, the identification and validation of biomarkers of cardiovascular disease in NHPs could significantly inform the management of cardiovascular disease in PLWH.
